# Preventing Overdoses Involving Stimulants: The POINTS Study Protocol

**DOI:** 10.21203/rs.3.rs-3993989/v1

**Published:** 2024-03-12

**Authors:** Jaclyn Hughto, Josiah Rich, Patrick Kelly, Stephanie Vento, Joseph Silcox, Madeline Noh, David Pletta, Earth Erowid, Fire Erowid, Traci Green

**Affiliations:** Brown University School of Public Health; Rhode Island Hospital; Brown University School of Public Health; Brown University School of Public Health; Brandeis University; Brown University School of Public Health; Brown University School of Public Health; Erowid Center; Erowid Center; Brandeis University

**Keywords:** stimulants, cocaine, crack, methamphetamine, fentanyl, opioids, overdose, mixed methods, drug checking, intervention development

## Abstract

**Background:**

In recent years, overdoses involving illicit cocaine, methamphetamine, and other stimulants have increased in the U.S. The unintentional consumption of stimulants containing illicit fentanyl is a major risk factor for overdoses, particularly in Massachusetts and Rhode Island. Understanding the drug use patterns and strategies used by people who use stimulants (PWUS) to prevent overdose is necessary to identify risk and protective factors for stimulant-involved overdoses. Mixed-methods research with people who distribute drugs (PWDD) can also provide critical information into the mechanisms through which fentanyl may enter the stimulant supply, and the testing of drug samples can further triangulate PWUS and PWDD perspectives regarding the potency and adulteration of the drug supply. These epidemiological methods can inform collaborative intervention development efforts with community leaders to identify feasible, acceptable, and scalable strategies to prevent fatal and non-fatal overdoses in high-risk communities.

**Methods:**

Our overall objective is to reduce stimulant and opioid-involved overdoses in regions disproportionately affected by the overdose epidemic. To meet this long-term objective, we employ a multi-pronged approach to identify risk and protective factors for unintentional stimulant and opioid-involved overdoses among PWUS, and use these findings to develop a package of locally tailored intervention strategies that can be swiftly implemented to prevent overdoses. Specifically, this study aims to [1] Carry out mixed-methods research with incarcerated and non-incarcerated people who use or distribute illicit stimulants to identify risk and protective factors for stimulant and opioid-involved overdoses; [2] Conduct drug checking to examine the presence and relative quantity of fentanyl and other adulterants in the stimulant supply; and [3] Convene a series of working groups with community stakeholders involved in primary and secondary overdose prevention in Massachusetts and Rhode Island to contextualize our mixed-methods findings and identify multilevel intervention strategies to prevent stimulant-involved overdoses.

**Discussion:**

Completion of this study will yield a rich understanding of the social epidemiology of stimulant and opioid-involved overdoses in addition to community-derived intervention strategies that can be readily implemented and scaled to prevent such overdoses in two states disproportionately impacted by the opioid and overdose crises: Massachusetts and Rhode Island.

## Background

In recent years, the United States has seen a drastic increase in the number of overdose deaths involving illicit stimulants such as powdered and crack cocaine and methamphetamine.^1–5^ According to national surveillance efforts, the majority of stimulant-involved overdose deaths also involved the synthetic opioid fentanyl.^6–11^ Illicitly manufactured fentanyl (herein referred to as fentanyl) has proliferated in the drug market and is highly lethal, especially for people with no or low tolerance to opioids.^4,12^ While the spike in stimulant and opioid-involved overdoses has been observed nationally, the Northeast region of the U.S. has been disproportionately affected by this crisis.^13^ Geographically-targeted research is needed to understand the local drivers of the stimulant and opioid-involved overdose epidemic.

Massachusetts and Rhode Island have some of the highest rates of fatal overdoses per capita, and surveillance data show an upward trend in fatal and non-fatal stimulant and opioid-involved overdoses in these states.^13^ In 2018, Massachusetts and Rhode Island had the 3rd and 5th highest age-adjusted rates of drug poisoning involving cocaine per 100,000 people, respectively.^14^ Further, since 2014, 9 in 10 deaths in Massachusetts that involved a stimulant also involved an opioid.^15^ Similarly, the proportion of overdose deaths in Rhode Island, where cocaine was detected in the deceased, increased from 33% in 2014 to 49% by 2022.^16^ Notably, in 2021, fentanyl was present in 75% and 85% of the overdose deaths involving cocaine in Rhode Island and Massachusetts, respectively,^13^ suggesting that exposure to fentanyl is the primary driver of fatal cocaine-involved overdoses in the overdose hotspot states of Massachusetts and Rhode Island. In light of these data, understanding drug use patterns (e.g., intentional vs. unintentional co-use of fentanyl and stimulants) and strategies to prevent overdose among people who use stimulants (PWUS) is a necessary step toward addressing the overdose epidemic.

While post-mortem data show an increase in overdoses involving both stimulants and opioids,^15,16^ the extent to which these substances are intentionally or unintentionally consumed by PWUS is under-studied, as it is not possible to survey individuals who are deceased about the drugs they intended to use prior to overdosing. Polysubstance use, such as the intentional use of opioids and stimulants, is a well-documented risk factor for overdose.^15,17,18^ However, research finds that people who intentionally use fentanyl or heroin alone or together with stimulants are generally aware of the risk of consuming highly potent fentanyl and employ harm reduction practices to prevent fatal overdoses,^19,20^ whereas people who only use stimulants may not.^19^ Indeed, formative research conducted by our team found that people who primarily use cocaine and unwittingly consume fentanyl are among the highest risk of experiencing an unintentional overdose and are the least prepared group of people who use drugs (PWUD) to appropriately respond to an opioid-involved overdose.^19^ For example, in our rapid assessment research in Massachusetts, we found that people who use cocaine and have no history of opioid use were less likely than those with a past or current history of opioid use to be equipped to recognize the signs and symptoms of an opioid overdose, call 911, carry naloxone, or be trained to administer naloxone.^19^ These individuals also reported worrying that they might harm a person by administering naloxone, and several reported that they witnessed a fatal overdose due to a lack of intervention on the part of ill-equipped bystanders.^19^ Our formative research highlights the urgent need to expand drug-checking services for the stimulant supply and to increase PWUS’ awareness of fentanyl in stimulants and their capacity to save lives by recognizing an opioid overdose, providing naloxone, and utilizing supportive services like 911 and the Never Use Alone overdose prevention hotline to reduce their risk for opioid-involved overdose.^21^

Examining the distribution of substances by people who distribute drugs (PWDD) is warranted^22^ as such research can also provide insights into the changing drug supply and associated risk proliferation. While the co-use of substances may be intentional, fentanyl contamination in the stimulant supply is increasing and may result in unintentional consumption, overdose, and death.^23–30^ Indeed, news stories have documented instances of bag mix-ups, in which PWDD have unintentionally given their clients fentanyl instead of cocaine, leading to their inadvertent consumption of fentanyl and overdose deaths,^31,32^ but a dearth of research has explored the frequency of and reasons why these bag mix-ups occur. Other unintentional pathways have been documented in the literature, including the cross-contamination of fentanyl and cocaine when products are cut and packaged on the same surfaces.^33,34^ Further, in research conducted by our team and others, PWUD reported various theories about why PWDD may intentionally adulterate the drug supply with fentanyl.^19,22,34–36^ These theories include beliefs that fentanyl is entering the supply to get people “hooked” on a more addictive substance in order to make more money.^22,34–36^ Given that a dearth of research has examined how and why fentanyl enters the stimulant supply specifically, surveying and interviewing people who use stimulants is needed to explore whether similar theories are endorsed in relation to the contamination of the stimulant supply. Notably, however, people who use stimulants may have limited knowledge of the intentions and behaviors of PWDD, particularly those who are higher up in the drug distribution hierarchy (e.g., suppliers, manufacturers, traffickers).^22^ Thus, conducting mixed-methods research with those involved at different levels of drug distribution who are actively involved in distributing in the community or who are incarcerated for drug distribution and manufacturing could help to better understand the changing nature of the stimulant supply and risk factors for stimulant and opioid-involved overdoses.

Drug checking can also help to answer questions about the consumption of substances and provide evidence about contamination and the relative potency of the stimulant supply.^22,34,37,38^ Drugs seized for criminal prosecution provide a small, selective view of the drug supply that overlooks information that may be useful for PWUD and public health. Further, typical forensic drug testing procedures divorce the individual from the substance, which severs all critical knowledge about the consumers’ experience using the substance. The loss of these data and the chance to inform and possibly recommend safer-use strategies for PWUD are some of the key motivations for the establishment of drug-checking services.^38–40^ Collecting and testing drug samples from PWUD in community settings and evaluating their knowledge about the contents of and experience using the product can provide critical insights into the drug supply.^37,41^ Moreover, these procedures have been readily employed and evaluated in community settings in Massachusetts by members of our team and have been found to not only be feasible and acceptable by community organizations and the clients they serve but have also been shown to generate essential, actionable data.^37,41^

Years of research and programmatic work to address the opioid crisis have led to the development of effective, multilevel strategies to address fatal and non-fatal overdoses among people who intentionally use opioids.^42^ While it is possible that these intervention tools can be leveraged to prevent stimulant and opioid-involved overdoses among people who use stimulants, interventions that are implemented without the buy-in of community members with lived expertise and those charged with implementing such strategies are less likely to be effective.^43^ Moreover, although the stimulant and opioid-involved overdose epidemic is a national problem,^13^ this problem is driven by regionally specific historical events and contextual factors that require local solutions.^44–48^ Thus, collaborating with local stakeholders involved in overdose prevention and response in high-risk geographic areas is essential to reducing stimulant and opioid-involved overdoses in the most affected areas of the U.S.

The *POINTS: Preventing Overdoses Involving Stimulants* Study aims to 1) conduct mixed-methods research with people who use stimulants and distribute drugs to identify risk and protective factors for stimulant and opioid-involved overdoses; 2) collect remnant drug samples to qualitatively and quantitatively characterize the drug supply and explore the presence of fentanyl and other adulterants relative to reported use patterns; and 3) collaborate with stakeholders from across the overdose prevention and response continuum to develop feasible, acceptable, and scalable multilevel strategies to prevent stimulant and opioid-involved overdoses in high-risk regions of Massachusetts and Rhode Island. Findings from this study, which include epidemiological outcomes, qualitative narratives, drug-checking results, and intervention development packages, will be presented back to the local communities, state-wide agencies, and federal agencies. The packages of locally tailored intervention strategies that we collaboratively develop will include the identification of resources (e.g., financial, change agents) to facilitate the swift implementation of interventions in Massachusetts and Rhode Island. While these strategies will be geographically tailored, we will illustrate ways in which the planned interventions can be adapted to meet the needs of other high-risk areas throughout the U.S. This approach will ensure both the feasibility and sustainability of our planned interventions in Massachusetts and Rhode Island as well as maximize the investment of federal overdose prevention funds by enabling this research to inform overdose prevention and response activities nationwide.

## Methods

**Figure 1.**
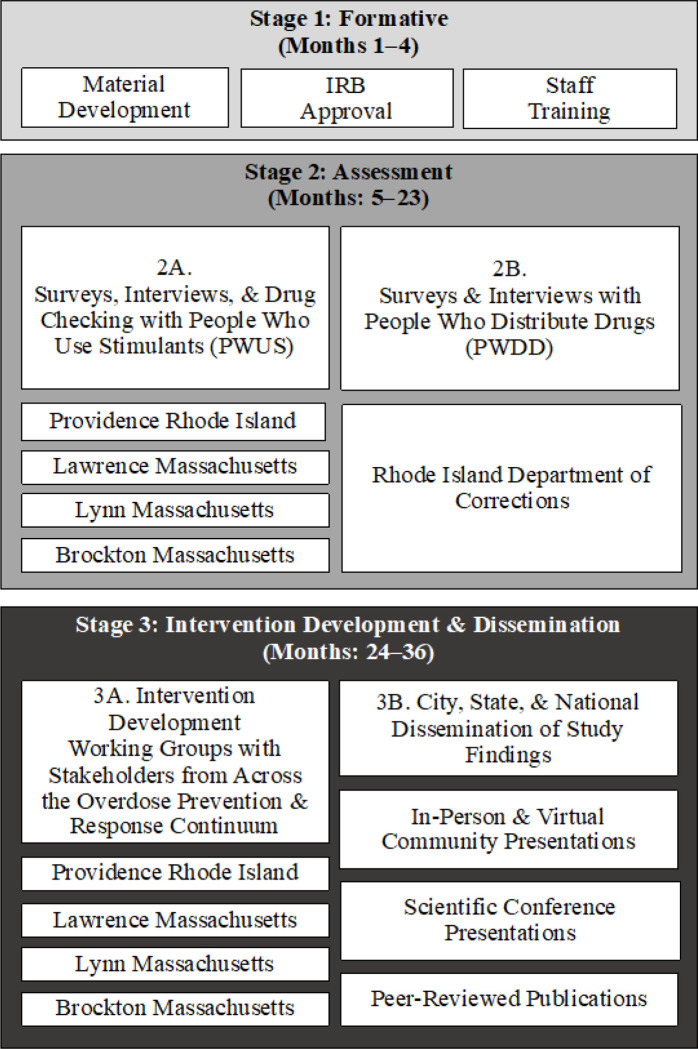
The multi-stage POINTS study design

This mixed-methods study follows a sequential intervention development approach involving several stages over 36 months (Figure 1). The Formative Stage (Stage 1: months 1–4) consists of study start-up activities, including material development, IRB approval, and staff training. The Assessment Stage (Stage 2: months 5–23) involves the collection of survey data and in-depth qualitative interview data with people who use stimulants (PWUS) and people who distribute drugs (PWDD) in Greater Providence, Rhode Island and Lawrence, Lynn, and Brockton Massachusetts and the Rhode Island Department of Corrections (Aim 1) and drug checking (Aim 2). The Intervention Development & Dissemination Stage (Stage 3: months 24–36) involves the formation of stakeholder working groups and the completion of 4 workshops each (16 total) in Providence, Rhode Island (RI); Lawrence, Massachusetts (MA); Lynn, MA; and Brockton, MA to interpret the findings from Aims 1 and 2 and develop multilevel intervention strategies to prevent stimulant and opioid-involved overdoses (Aim 3); as well as multi-modal dissemination efforts, including numerous in-person and virtual presentations on study findings to city, state, and national audiences, national and international scientific conference presentations, community-focused dissemination materials, and peer-reviewed publications.

### Data Collection Sites & Community Partners

Drawing on overdose surveillance data^13^ and our prior research with PWUD in Massachusetts and Rhode Island,^19,36,49–52^ we selected 4 locations where fatal stimulant and opioid-involved overdoses were concentrated from which to recruit PWUS and stakeholders. Locations include Providence, RI; Lawrence, MA; Lynn, MA; and Brockton, MA. Statewide fatal overdose rates in 2019 were 29.0 per 100,000 in MA and 29.1 per 100,000 in RI. The 2019 fatal overdose rate in each study location exceeded these rates (see Table 1).^16,53–55^

Although we recognize that many PWUS may also have a history of drug distribution and many PWDD may also use drugs, in an effort to reach PWDD who are higher up in the drug distribution hierarchy, we also selected The Rhode Island Department of Corrections (RIDOC) as an additional site through which to recruit PWDD (see additional details below).

### Stage 1 Formative Procedures.

The first stage involves the development of the study protocol, recruitment materials, quantitative survey, qualitative interview guides, and hiring and training of study staff. During this stage, Institutional Review Board (IRB) approval is obtained, and institutional agreements between the coordinating site, Brown University, and the collaborating sites, Brandeis University and Rhode Island Hospital, are obtained. Internal approval from the RIDOC Medical Research Advisory Group, the internal RIDOC research approval board, is also obtained in Stage 1. As a federally-funded study, a Certificate of Confidentiality is also provided by the Centers for Disease Control and Prevention (CDC), which provides additional protections for research participants by prohibiting researchers from disclosing identifying information as part of any federal, state, or local civil, criminal, administrative, legislative, or other action, suit, or proceeding, or to be used as evidence, including by subpoena.

### Stage 2 Assessment Procedures.

In Stage 2, we employ 3 different methods to rigorously collect data and evaluate risk and protective factors for stimulant and opioid-involved overdoses. These methods include [1] Quantitative Surveys: Conducting up to 260 surveys (n=65 per site) with PWUS in Greater Providence, Lawrence, Lynn, and Brockton and 30 surveys with PWDD incarcerated at RIDOC; [2] Qualitative Interviews: Completing in-depth qualitative interviews with up to 90 of the PWUS who are surveyed in the 4 regions (~22 per site) and with all 30 PWDD who complete surveys at RIDOC; and [3] Drug Checking: All non-incarcerated participants are invited to donate their drug trash (e.g., old baggies, cookers, cottons, glass pipes, stems) for on-site drug testing using fentanyl test strips and a Fourier Transform Infrared Spectroscopy (FTIR) machine. Following on-site drug-checking activities, the donated samples are sent to the DrugsData lab for confirmatory testing (see drug-checking methods below for additional details).

#### Stage 2. Inclusion/Exclusion Criteria.

The participant sample for this stage includes [1] PWUS: people who use stimulants (e.g., powdered and crack cocaine, methamphetamine, or street-obtained prescription stimulants), recruited from the 4 locations in MA and RI; and [2] PWDD: people who have a history of distributing/manufacturing drugs (e.g., including opioids and/or stimulants), recruited from RIDOC.

PWUS who are recruited in the 4 community sites are eligible to participate if they are: 1) 18 years of age or older; 2) able to speak and understand English or Spanish; 3) used an illicit stimulant in the past 30 days; 4) live in or spend the majority of their time in one of the 4 geographic areas: Greater Providence, RI; Lawrence, MA; Lynn, MA; or Brockton MA; and 5) willing and able to provide informed consent.

PWDD who are recruited from RIDOC are eligible to participate if they are: 1) 18 years of age or older; 2) speak and understand English or Spanish; 3) currently incarcerated at RIDOC; 4) currently or previously sentenced for drug distribution or manufacturing charges; 5) have been incarcerated for less than 3 years of their current sentence; and 6) willing and able to provide informed consent.

#### Stage 2. Recruitment.

Two different sampling approaches were used to recruit PWUS at the 4 community sites and PWDD at RIDOC.

##### PWUS: Community-Based Referrals.

Drawing on our success recruiting PWUD for rapid mixed methods studies in Massachusetts,^19,49–51,56–62^ the POINTS study uses a modified respondent-driven sampling approach to recruit PWUS at the 4 community sites.^57,62^ Respondent-driven sampling is a network-based sampling method that starts with a convenience sample of initial participants (herein referred to as “seeds”) and uses small incentives (e.g., $5 cash, which is modest enough to not engender coercion) to recruit the networks of the seed participants.^63^ Participants receive coupons with unique identification numbers for themselves as well as their recruits.^63^ For the present study, using CDC State Unintentional Drug Overdose Reporting System (SUDORS) data,^13^ we explored the demographics of individuals who died of stimulant-involved opioid overdose in each of the 4 regions in 2020 and sought to identify seed participants with similar characteristics as the decedents. This included a higher proportion of males, Black and Hispanic individuals, and individuals who were housed. Our formative work in the 4 locations also included environmental scans and ethnographic mapping to identify community partner organizations and geographic locations to maximize the successful use of respondent-driven sampling.^19,36,50,57,64^ Drawing on our formative research with PWUS that identified differences in overdose risk by substances used,^19^ we identified seeds according to their past and current substance use history in order to recruit individuals who 1) currently use stimulants and have no history of intentional opioid use, 2) currently use stimulants and have a history of intentional opioid use, and 3) currently use stimulants and opioids. The seeds are identified in collaboration with community partner organizations in each city that serve PWUS. These partners include staff at harm reduction organizations, primary care and outpatient substance use treatment settings, and faith-based organizations. By working with community partners who have close ties to PWUS in their community, we are able to readily identify individuals who are well known within the community and have an extensive network of PWUS whom they can recruit to participate in the study.

Once the initial seeds are selected and complete the survey and interview, they are given four coupons with a unique code and asked to refer up to four people that they know who might be eligible and interested in participating in the study (i.e., “sprouts”). Eligible sprouts have two weeks to return the coupon and complete the one-time study visit. Following completion of data collection, sprouts are given three coupons and asked to refer additional sprouts who would be a good fit for the study. When coupons are returned by a sprout, the participant who referred the sprout receives $5 cash (up to three referrals; $15 cash). This process continues until we reach our target sample size of PWUS in each location.

##### PWDD: RIDOC Correctional Facility Referrals.

PWDD are recruited to participate in the study while incarcerated. RIDOC staff provide the study team with a list of incarcerated individuals who have been sentenced for drug distribution or manufacturing. Study staff then mail the potential participants a study information card with a general description of the study. To protect participant safety and confidentiality, our recruitment language and materials focus on “knowledge of the drug supply” rather than “drug distribution” specifically. The card also notes that the study team will be requesting to meet with them at RIDOC in the coming weeks and includes a study phone number so that potential participants can call us to learn more about the study or opt out of participation in advance of our arrival. Study staff then travel to the RIDOC campus during the dates and times approved by the Warden and request to meet with potential participants. Potential participants who have received an information card and are willing and able to meet with us are then brought into a private area to learn more about the study. If the individual is interested in participating, they are screened for eligibility. Staff then conduct an informed consent process with eligible participants, and those who consent to participate are enrolled in the study, and data collection subsequently begins.

#### Stage 2 Data Collection:

##### Quantitative Survey.

Quantitative surveys are administered to all PWUS enrolled at the 4 community sites and all PWDD enrolled at RIDOC. Prior to conducting the survey, all participants undergo an informed consent process. We obtained a waiver of written consent to allow participants in the community to provide verbal consent. Incarcerated participants provide written consent per RIDOC’s guidelines.

Both surveys are programmed into Qualtrics, a secure web-based survey administration tool, and administered by study staff. Time to complete the survey is about ~30–45 minutes for PWUS in the community. A shorter, ~20–30 minute survey is administered to RIDOC participants due to institutional time constraints and our study design, which involves the collection of survey and interview data from all incarcerated participants. Using measures previously developed and tested in our past research and other studies with PWUD, the structured survey assesses sociodemographic characteristics, substances used, physical and mental health conditions and treatment use, opioid overdose history, knowledge of naloxone and overdose prevention policies, attitudes toward and experience with treatment (community participants only), awareness of contamination of the drug supply, and more.^19,36,50,51,56,62,65,66^ We also developed new items to assess stimulant toxicity or overamping. The survey administered to RIDOC participants drew on adapted measures from prior research,^67–70^ including arrest, offense, incarceration history, and the intentional (i.e., drug cutting) mixing or adding other substances to the illicit drug and newly developed items to assess the unintentional mixing or distribution of fentanyl into illicit stimulants and other drugs and drug supply-related harm reduction practices. Incarcerated participants receive $20 in commissary funds, and community participants receive $20 cash for completing the quantitative survey.

##### Qualitative Interviews:

In-depth, qualitative interviews are administered to a subset of PWUS enrolled at the four community sites and all PWDD at RIDOC. Specifically, approximately one-third of community PWUS are invited to complete an interview. Based on participants’ survey responses, study staff are trained to offer interviews to individuals who are diverse in terms of age, gender, race/ethnicity, housing, SES, and primary type of substance used (e.g., cocaine, meth, counterfeit stimulant pills, opioids) and/or have unique or extensive patterns of use, overdose experiences, and experiences accessing harm reduction and treatment services. All incarcerated participants will complete an interview. For both samples, the interviews take approximately 30–45 minutes. Study staff utilize a semi-structured interview guide, which seeks to probe in greater depth about the same domains assessed in the quantitative survey. All participants are compensated $20 for completing the qualitative interview (commissary funds for incarcerated participants and cash for community participants).

All interviews are audio-recorded and professionally transcribed, after which the audio files are deleted. Participants are reminded to refrain from sharing personally identifying information, including names of individuals or businesses, during the interview. Incarcerated participants are specifically encouraged to discuss their general knowledge about fentanyl in the drug supply and other potentially criminalizing questions so as not to incriminate themselves by sharing direct experiences. Personally-identifying information that is inadvertently shared is removed from the electronic transcript by study staff. Following each interview, study staff write detailed memos cataloging emerging themes and key observations.

##### Drug Checking:

Our drug-checking procedures are derived from prior innovative work conducted by members of our team as part of the Massachusetts Drug Supply Data Stream project.^37^ Specifically, drug checking is only performed with samples gathered from community participants (i.e., samples are not collected from incarcerated participants). PWUS are invited to provide drug “trash,” including drug packaging (e.g., baggies) or works (e.g., pipes, cookers) with drug residue for the purposes of drug checking. No syringes are collected. The drug trash sample is inspected by study staff for visible residue, stored in plastic bags, and cataloged with the time and date of acquisition and a unique study ID number. A brief 15-item Qualtrics survey posing questions about the sample (i.e., presumed content of the sample, city where the packaging was obtained, purchase price (if known), preparation and use experiences, and overall impression of the quality and content of the sample) is then administered to the participant by a member of the study team. Participants are invited to provide up to three drug samples and are reimbursed $5 cash for each sample they provide.

The drug samples are tested by a trained drug-checking team led by the senior author.^37^ First, the drug-checking team gathers in a private room in accordance with our drug-checking standard operating procedures developed based on established safety protocols.^71–73^ Example safety measures include the requirement that when conducting sample measurement and scanning, operational technicians must wear nitrile gloves, which should be changed on a regular basis (every 30 to 60 minutes) whenever they come into contact with a substance or if the gloves tear.^37^ Study staff are trained to always remove gloves and wash hands before touching their face, touching doorknobs, using the restroom, eating, drinking, or leaving the sample scanning area.^37^ The contents of the drug packaging are scraped onto a scanning plate and scanned via compact FTIR (Fourier Transform InfraRed spectroscopy), and the results are recorded. A portion of the scanned sample is then diluted in 5ml sterile water and tested using the fentanyl immunoassay test strips (BTNX), and the results are recorded. Any remnant drug, packaging, or water is discarded using a Deterra drug disposal bag, and the FTIR is cleaned using an isopropyl alcohol/alcohol prep pad.

Notably, FTIR and fentanyl test strips are employed before confirmatory lab testing as these techniques are less expensive, faster, and non-destructive and can be conducted by non-chemists.^71^ However, these techniques cannot be limited in their ability to detect low concentrations of key substances, like fentanyl; thus, confirmatory lab testing is performed.

Across sites, 25–100% of the samples in each geographic location are selected for confirmatory GC/MS (Gas Chromatography/Mass Spectrometry) lab testing. The decision to send a portion of the samples for confirmatory testing is based on funding and the availability of sufficient drug residue following FTIR and fentanyl test strip testing. In instances where a subset of viable samples is prioritized for confirmatory testing, samples are selected if the participant reports an adverse experience with the drug or the sample appears to contain a unique cut product. Viable samples that are sent out for lab confirmation are packaged in a secured mylar envelope and mailed to our confirmatory testing partner DrugsData. DrugsData, a project of Erowid Center, contracts with a Drug Detection Laboratories, which has special permissions from DEA permitting testing of anonymous mailed-in samples of psychoactive substances for DrugsData. These samples are destroyed following testing. Results from all lab-tested substances are published publicly at www.drugsdata.org.

#### Stage 2 Data Analysis:

##### Quantitative Analyses:

Data from the surveys with PWUS in the community and PWDD at RIDOC is downloaded from Qualtrics, cleaned, and collated into a single dataset. Separate analyses are then performed for the samples of PWUS and PWDD. Descriptive statistics (frequencies, means) are used to summarize the frequency of all variables overall and by region. For the PWUS sample, bivariate statistics (t-tests/χ2) are used to explore differences in all study variables according to current and past substance use history (i.e., people who only use stimulants; people who only use stimulants but have a history of opioid use; and people who use both stimulants and opioids).

##### Qualitative Analyses:

Transcriptions and memos are checked for accuracy and uploaded into Dedoose, a secure, cloud-based qualitative data management program.^74^ The study team utilizes integrated thematic analysis, which pairs deductive codes aligned with the semi-structured interview guide with inductively created codes based on emergent patterns in the data to create a core codebook. First, a preliminary codebook is created consisting of deductive codes from the semi-structured interview guide and inductive codes generated through open-coding of transcripts from Greater Providence, Rhode Island—the first data collection site. The open coding process identifies concepts that are otherwise not captured by deductive codes. Inductive and deductive codes are then organized by like-concept and hierarchically. Coders then independently apply the codebook to a set of transcripts and engage in discussions to refine the coding process and codebook to determine that further revisions are not necessary. Coding of all transcripts then occurs by a trained qualitative analysis; transcripts are not double coded, but code applications are discussed through regular team meetings to ensure consistency in code application. This process repeats following the collection of qualitative data in each recruitment location to add inductive codes that are region-specific. For each site, once the codebook is finalized, independent coders apply the codes to the transcripts for the site. Within- and across-case analyses are then used to examine data within individuals and across study locations.

##### Drug Checking Analyses:

FTIR and confirmatory drug testing data are descriptively summarized (means, frequencies), and the two methods are statistically compared (t-tests/χ2) to determine the reliability of the FTIR results for the active drug components and of the fentanyl test strip for fentanyl detection for instances where laboratory data are available. Drug content information from two or more testing procedures informed analysis of fentanyl “contamination” (i.e., testing detected presence of fentanyl in a drug suspected, bought or otherwise expected to be a drug other than fentanyl). Drug-checking findings are also triangulated against participant self-report using bivariate analyses (t-tests/χ2). Data are summarized by recruitment region and later combined, stratified, and assessed for global differences by location (χ2). For all statistical tests, alpha is determined at p<0.05.

### Stage 3 Procedures: Intervention Development Stage.

Stage 3 involves the utilization of working groups composed of regional leaders across the overdose prevention and response continuum to develop locally tailored yet scalable interventions in each of the 4 high-risk communities. It consists of three phases: [1] pre-meeting analytics; [2] stakeholder workshops; and [3] post-meeting dissemination.

#### Stage 3. Inclusion/Exclusion Criteria.

Eligible working group members are: 1) 18 years of age or older; 2) can read, write, speak, and comprehend English; 3) are involved in stimulant use or overdose prevention or response activities or have a history of drug use; 4) live or work in the recruitment region of focus (i.e., Greater Providence; Lawrence; Lynn; Brockton); and 5) are willing and able to provide informed consent.

#### Stage 3. Recruitment.

Up to 10 local leaders in each of the geographic areas are recruited to participate in 4 community-based intervention development workshops. The survey completed by PWUS in Stage 2 asks participants to name local leaders within their community who help to keep PWUD safe, and these responses inform the purposive approach to recruiting stakeholders. Additionally, the study team works with community partners to recruit multi-disciplinary stakeholders, including but not limited to people with a history of stimulant use; harm reduction workers; primary care, emergency department, and substance use treatment providers; recovery coaches; law enforcement personnel, emergency medical service providers, and other first responders; pharmacists; religious leaders; and housing, food, and social support service leaders. Individuals were selected as 1) they are local leaders within the region; 2) are heavily involved in one or more stages of the overdose prevention and response continuum; and 3) likely have the capacity, respect, and influence to readily implement one or more of the collaboratively developed intervention strategies following the completion of the workshops.

#### Stage 3 Intervention Development

**Figure 2.**
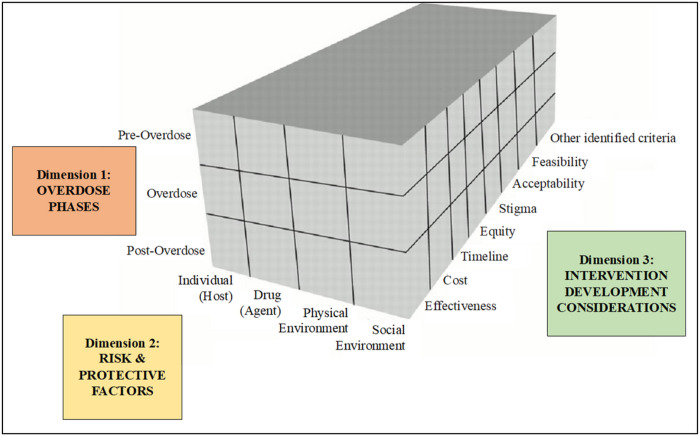
Adapted Haddon Matrix of Risk and Protective Factors and Considerations for Developing Interventions to Prevent Stimulant and Opioid-Involved Overdoses.

The intervention development process is guided by the Haddon Matrix, a heuristic used in public health injury prevention research that considers the multi-level risk and protective factors before, during, and after an injury or death.^75–78^ As the first group of researchers to adapt and apply this model to the stimulant-involved overdose epidemic, our application of the Haddon Matrix model includes 3 dimensions of overdose risk and response (see Figure 2). The first dimension considers the 3 phases or timing of a given factor in relation to an overdose injury event: *Pre-Overdose, Overdose*, and *Post-Overdose*. The second dimension considers the level at which risk and protective factors related to stimulant-involved overdoses occur: the *individual (host), drug (agent), physical environmental* and *social environment*. Drawing on prior adaptations of the original Haddon Matrix,^77^ we also consider a third dimension comprised of important decision-making components alongside the causal factors of the matrix, such as *cost, feasibility, acceptability, e*quity, and *timeline*. For the current protocol, this third dimension focuses on the various factors that should be considered when developing intervention strategies to prevent and respond to stimulant and opioid-involved overdoses.

During Stage 3, we use the Haddon Matrix both as an analytic framework to organize the risk and protective factors identified via the Stage 2 mixed-methods data collection as well as a framework to guide the intervention development process as part of the Stage 3 workshops.

As shown in Table 2, the intervention development process consists of 3 phases with their own procedures, interim analyses, and outputs.

##### Phase 1. Pre-Meeting Analytics.

In preparation for the working groups, a series of analyses are conducted to elucidate local risk and protective factors for stimulant and opioid-involved overdoses.

#### Stage 2 Analyses.

The site-specific quantitative survey, qualitative interview, and drug-checking data are analyzed, and local risk and protective factors for stimulant and opioid-involved overdoses are identified and summarized alongside site-specific fatal overdose data drawn from state SUDORS data.

#### Working Group Member Survey.

Prior to the first workshop and after the final workshop, all stakeholders complete a brief quantitative survey on Qualtrics that collects background information on stakeholders’ knowledge of, experience with, and current involvement in stimulant, opioid, and polysubstance overdose prevention and response activities; suggestions for intervention strategies; and perceptions of facilitators and barriers to addressing stimulant-involved overdoses in their local area.

##### Phase 2. Stakeholder Workshops:

This study utilizes an efficient approach to intervention development that minimizes participant burden and maximizes engagement. This includes holding 4 working group meetings in 4 cities over 9 months. Each working group meets once a week for 4 weeks over one month (4 meetings total). Each meeting lasts approximately 1.5 hours. The meetings are hosted in person at a central location in each region and are facilitated by the study investigators. Although all working group members are strongly encouraged to attend in person to facilitate participation in all meetings, we also offer hybrid Zoom participation.

### Phase 3. Post-Meeting Analytics and Dissemination

We will conduct descriptive analyses (means, frequencies) of the quantitative variables contained in the stakeholder surveys. Open-ended questions in the stakeholder survey will be coded using a thematic analysis approach. Findings from the stakeholder workshops will be synthesized and iteratively packaged and re-packaged until a final set of proposed intervention strategies is created for each of our 4 geographic regions. Information about proposed intervention strategies will be shared across various outlets, including at local, state, and national presentations and academic conferences, and will also be disseminated in written community-facing infographics and peer-reviewed journals.

## Discussion

Local and national efforts to reduce the overdose epidemic are hampered by a lack of data about the drug supply, how people use drugs, and community and stakeholder insights into the feasibility and acceptability of intervention strategies. The POINTS study addresses these gaps by innovatively and concurrently surveying and interviewing PWUS and PWDD in community and correctional settings as a means of identifying multiple risk pathways for (and opportunities to prevent) stimulant-involved overdoses in high-overdose-risk regions of the U.S. The POINTS study will also be the first to triangulate mixed-methods findings gathered from incarcerated and non-incarcerated PWUS and PWDD against real-time drug-checking services to determine whether drug samples provided by people who use and distribute stimulants and other drugs contain the substances believed to be in the sample. Findings will help yield objective data on the quality and potency of the local stimulant supply in four overdose hotspot regions of Massachusetts and Rhode Island.

The POINTS study also seeks to shift overdose response paradigms by using a data-informed and collaborative approach to working with local communities to develop innovative, geographically tailored and scalable solutions to the stimulant and opioid overdose crisis. Although there are strategies that can be borrowed from the opioid-overdose response literature, the rise in fatal stimulant and opioid-involved overdoses underscores that the most at-risk people who use stimulants are not benefiting from the extensive opioid-overdose prevention and response activities taking place in some of the most affected communities. Thus, to address the stimulant and opioid-involved overdose crisis, there is a need to expand current prevention activities to address the needs of PWUS. Our mixed-method research to gather information about overdose risk and protective factors with PWUS and PWDD will provide essential data to inform our collaborative efforts with local leaders from across the overdose prevention and response continuum to identify novel strategies to prevent stimulant and opioid-involved overdose. Since identified strategies will only be effective if the people tasked with implementing these strategies are convinced of the utility of the strategies and have the motivation to implement these strategies, our plan to work with local community stakeholders aims to ensure that the intervention packages we collaboratively develop can be swiftly and feasibly implemented in communities of interest to reduce stimulant and opioid-involved overdoses and achieve maximum public health impact in Massachusetts, Rhode Island, and beyond.

## Figures and Tables

**Table 1.** Characteristics of community recruitment regions/sites

Region / Recruitment Site	Population Size	County	2019* Fatal Overdose Rate per 100,000 People
Lawrence, Massachusetts	80,028	Essex, North of Boston	67.5
Lynn, Massachusetts	94,299	Essex, North of Boston	58.7
Brockton, Massachusetts	95,708	Plymouth, South of Boston, North of Providence	52.2
Greater Providence, Rhode Island Includes: Central Falls, Providence, Pawtucket, East Providence, Cranston	400,642	Providence	Central Falls 56.8, Providence 38.8, Pawtucket 43.6, Cranston 23.6, East Providence 23.4

* Year of most recently available data preceding data collection in each location

**Table 2.** Collaborative, Multi-Phase Intervention Development Workshops with Local Stakeholders

PHASE	ACTIVITIES
**1. Pre-Meeting Analytics**	Findings from the pre-meeting analyses will be tabulated and prepared for working meetings and ultimately dissemination
**2. Stakeholder Workshops**	-
Workshop 1: Data Sharing	Review of surveillance data and stage 2 findings, including local risk and protective factors for stimulant and opioid-involved overdoses.
Workshop 2: Strategy Generating	Collaborative identification of locally tailored stimulant and opioid-involved overdose prevention intervention strategies that are responsive to the localized risk and protective factors presented in workshop 1.
Workshop 3: Strategy Evaluation	Multi-step assessment and discussion of the perceived feasibility, acceptability, efficacy, cost, time-to-implement, and equity of each intervention strategy identified in workshop 2.
Workshop 4: Strategy Packaging and Implementation Preparation	Review of the short-term facilitators and barriers to intervention implementation and identification of “change agents” who can champion the implementation of each locally tailored intervention strategy in the region.
**3. Post-Meeting Analytics and Dissemination**	Local, State, & National Presentations: Compilation of study findings and presentation to various community audiences, with the goal of communicating risk and protective factors and encouraging the uptake of the collaborative, community-developed, and data-informed, locally tailored intervention strategies. Executive Summaries: Distilling of findings into brief reports highlighting key study findings by recruitment region. Scientific Conference Presentations: Dissemination of study findings via oral and poster presentations and invited talks at national and international conferences. Peer-Reviewed Publications: Dissemination of study findings via peer-reviewed publications in substance use, harm reduction, public health, and policy journals.

## Data Availability

All data and materials will be made publicly available in accordance with our data management plan. Interested persons should contact the first author to request access to the data and materials.
